# Changes in the Anti-Allergic Activities of Sesame by Bioconversion

**DOI:** 10.3390/nu10020210

**Published:** 2018-02-14

**Authors:** Tae-Dong Jung, Sun-Il Choi, Seung-Hyun Choi, Bong-Yeon Cho, Wan-Sup Sim, Han- Xionggao, Sang Jong Lee, Seon Ju Park, Dan-Bi Kim, Young-Cheul Kim, Jin-Ha Lee, Ok-Hwan Lee

**Affiliations:** 1 Department of Food Science and Biotechnology, Kangwon National University, Chuncheon 24341, Korea; lgtjtd@naver.com (T.-D.J.); docgotack89@hanmail.net (S.-I.C.); zzaoszz@naver.com (S.-H.C.); bongyeon.cho92@gmail.com (B.-Y.C.); simws9197@naver.com (W.-S.S.); xionggao414@hotmail.com (H.-X.); 2 STR Biotech Company, LTD., Chuncheon 24232, Korea; sj@strbiotech.co.kr or lsj@strbiotech.co.kr (S.J.L.); coco4649@strbiotech.co.kr (S.J.P.); 3 Korea Food Research Institute, Wanju-gun, Jeollabuk-do 55365, Korea; Kim.Dan-bi@kfri.re.kr; 4 Department of Nutrition, University of Massachusetts Amherst, Amherst, MA 01003, USA; yckim@nutrition.umass.edu

**Keywords:** atopic dermatitis, bioconversion, *Sesamum indicum* L., skin inflammation, anti-allergic

## Abstract

Sesame is an important oilseed crop, which has been used as a traditional health food to ameliorate the prevention of various diseases. We evaluated the changes in the anti-allergic activities of sesame by bioconversion. SDS-PAGE of non-fermented sesame proteins showed major allergen bands, while that of fermented sesame showed only a few protein bands. Additionally, we investigated the effectiveness of fermented sesame by bioconversion in tumor necrosis factor-α (TNF-α)- and interferon-γ (IFN-γ)-induced HaCaT cells. In HaCaT cells, fermented sesame inhibited the mRNA expression of interleukin-6 (IL-6) and interleukin-1β (IL-1β), thymus and macrophage-derived chemokine (MDC/CCL22), activation-regulated chemokine (TARC/CCL17), and intercellular adhesion molecule-1 (ICAM-1). Moreover, fermented sesame inhibited the activation of nuclear factor-κB (NF-κB) and signal transducer and activator of transcription 1 (STAT1). Fermented sesame exerts anti-allergic effects by suppressing the expression of chemokines and cytokines via blockade of NF-κB and STAT1 activation.

## 1. Introduction

Sesame (*Sesamum indicum* L.) is composed of about 50% lipid, 15% carbohydrate, 5% moisture, and 15% protein. The sesame seed is one of the most important oil crops cultivated in India, China, Sudan, and other Asian countries. In addition, the sesame seed has been used in traditional medicine for its anti-aging and anti-hepatotoxic effects. The oxidative stability of sesame oil is due to the presence of lignan compounds such as sesamol, sesamin, and sesamolin [1]. Several studies have reported the biological activity of lignan compounds, which include anti-lipid peroxidation [2], anticancer activity towards MCF-7 cells [3], and a protective effect against sepsis in rats [4]. However, sesame seed food allergies are becoming an increasingly recognized health issue, especially in developed countries, including the European Union [5]. The major allergen proteins in sesame have been identified in Canada and the United State: Ses i 1 (9 kDa) and Ses i 2 (7 kDa) are members of the 2S albumin family, while Ses i 3 (45 kDa) 7S globulin, Ses i 4 (17 kDa), and Ses i 5 (15 kDa) are members of the 2S oleosin family. Sesame allergen proteins have been identified by SDS-PAGE, mass spectrometry, and immunoblotting [6,7].

Atopic dermatitis (AD) is a common chronic inflammatory disorder of the skin. The recognition of food allergens by antigen-presenting cells in individuals with eczema could be an important mediator of food sensitization and food allergy.

Keratinocytes are the main epidermal cells that play a critical role in the occurrence of AD. Exposure of keratinocytes to tumor necrosis factor-α (TNF-α) and interferon-γ (IFN-γ) leads to the abnormal expression of cytokines, chemokines, and adhesion molecules, such as ICAM-1, which is considered to increase the infiltration of monocytes into the site of inflammation in the skin [8]. Macrophage-derived chemokine (MDC/CCL22) and thymus and activation-regulated chemokine (TARC/CCL17) and are members of the chemokine subfamily and are produced by various cell types, such as keratinocytes [9]. Since TARC is a ligand for CCR4, which is predominantly expressed in Th2 lymphocytes, it selectively controls the migration of Th2 lymphocytes into the site of inflammation [10]. MDC is constitutively produced by B cells, dendritic cells, keratinocytes, and macrophages. Thus, TARC and MDC play an important role in the development of skin diseases such as atopic dermatitis. TARC and MDC promoters contain STAT1 and NF-κB binding sequences, and these transcription factors may mediate the transcription of these genes [11].

Bioconversions such as fermentation, carried out by microorganisms and enzymes, may produce beneficial bioactive compounds. Apart from improving the bioavailability of nutrients, it has been hypothesized that fermentation regulates immunogenicity and possibly the reduction of allergenicity [12]. Our previous studies demonstrated that the used bioconversion process amplifies the antioxidant activity and bioactive compounds in sesame [13]. Many studies have demonstrated that soybean allergen degradation occurs during fermentation of soybean food [14,15]. However, an effect of sesame by bioconversion on the anti-allergic and anti-inflammatory activities has not been reported.

In this study, we evaluated the degradation of major sesame Ses i allergens by the bioconversion of sesame. Furthermore, we examined the anti-allergic activities of non-fermented and fermented sesame, TNF-α- and IFN-γ-induced production of cytokines (IL-1β and IL-6) and chemokines (TARC, MDC), and gene expression (IL-1β, IL6, ICAM-1, TARC, and MDC) via regulation of NF-κB and STAT1 signaling pathways in HaCaT cells.

## 2. Materials and Methods

### 2.1. Chemicals

Fetal bovine serum (FBS), Dulbecco’s modified Eagle’s medium (DMEM), and phosphate buffer saline (PBS, pH 7.4) were obtained from Gibco (Gaithersburg, MD, USA). Recombinant human TNF-α and IFN-γ were obtained from R&D System Inc. (Minneapolis, MN, USA). ELISA kits for human IL-1β, IL6, MDC/CCL22, and TARC/CCL17 were purchased from R&D System. Antibodies against IκBα, phospho-STAT1(Ser727), STAT1, phospho-NF-κB p65(Ser536) were purchased from Cell Signaling Technology (Beverly, MA, USA).

### 2.2. Sample Preparation

Sesame was harvested in August, 2014 and provided by the National Institute of Crop Science (Milyang, Korea). Sesame was fermented with *Lentinula edodes* using a fermentation system (STR biotech, Chuncheon, Korea). Briefly, *L. edodes* fungal mycelia were isolated from the mushroom fruit body and cultured in potato dextrose agar medium (PDA, Difco Laboratory, Detroit, MI, USA). The genetic identity of the fungus was confirmed by the Korean Center of Microorganisms (Seoul, Korea). The mycelia cultured on PDA media were inoculated in 50 mL of a liquid medium containing 2% glucose, 0.5% yeast extract, 0.5% soy peptone, 0.2% KH_2_PO_4_, 0.05% MgSO_4_, and 0.002% FeSO_4_. The experiments were conducted in 250 mL Erlenmeyer flasks at 28 °C for 5 days in a rotary shaker, and the resulting broth was used to seed the main liquid culture. A liquid culture medium containing sesame was treated with amylase and cellulase at 60 °C for 60 min for the enzymatic digestion of particulate materials containing carbohydrate. Subsequently, the culture mass was adjusted by bringing the pH to 6.0 with HCl, and sterilization was done in the autoclave. The main investigations with the liquid culture were started in a 5 L fermenter (working volume of 3 L) at 28 °C and 150 rpm by inoculating with the cultured mycelia (10%). After 7 days, the culture was treated with an enzyme mixture containing cellulase, hemicellulase, pectinase, glucanase, mannase, and arabinase at 50 °C for 60 min, for cell wall lysis. The enzyme-treated culture mass was then extracted at 90 °C after 1 h and freeze-dried to a solid material. Non-fermented and fermented sesame (500 mg) were mixed with 25 mL of PBS, and the mixture was sonicated for 60 min. The mixture was centrifuged at 3000× *g* for 15 min, and the supernatant was filtered through a 0.22 µm PVDF filter (Millex-HV, Millipore, Bedford, MA, USA).

### 2.3. Sodium Dodecyl Sulfate-Polyacrylamide Gel Electrophoresis (SDS-PAGE)

The molecular weight distribution was determined using SDS-PAGE according to a previously described method with some modifications [16]. A loading buffer (150 mM NaCl, 50 mM Tris-HCl, 1% Trizol, 0.1% SDS, 0.25% sodium deoxycholate, and 2-mercaptoethanol) was added to non-fermented and fermented sesame (30 µg total protein), and the mixture was boiled in a water bath for 5 min. The supernatant was electrophoresed using a 5% stacking gel and 15% separating SDS-polyacrylamide gel, after which the gel was stained with Coomassie blue.

### 2.4. Cell Culture and Cell Viability Assay

The human keratinocyte HaCaT cell line obtained from the cell line service (CLS) (Eppelheim, Germany) was maintained in DMEM supplemented with 10% FBS, penicillin (100 U/mL), and streptomycin (100 µg/mL) in a humidified, 5% CO_2_ incubator at 37 °C.

Cell viability was assessed using an XTT(2,3-*bis*-2-methoxy-4-nitro-5-sulfophenyl-2*H*-tetrazolium-5-carboxanilide) assay (Welgene, Seoul, Korea), performed according to the manufacturer’s instructions. After pre-incubation of HaCaT cells for 24 h in a 96-well plate (1 × 10^5^ cells/well), the cells were treated with non-fermented sesame (NS) and fermented sesame (FS) (50, 100, 200 µg/mL) and/or TNF-α (10 ng/mL)/IFN-γ (10 ng/mL) for 24 h. The XTT reagent was added, and the plate was incubated for 4 h. The absorbance was measured at 450 nm using a microplate reader (Molecular Devices, Sunnyvale, CA, USA).

### 2.5. Reverse Transcription-Polymerase Chain Reaction (RT-PCR)

HaCaT cells (1 × 10^6^ cells/well) were treated with TNF-α/IFN-γ or with medium alone in the presence or absence of NS or FS for 24 h. The total RNA was extracted using the Trizol reagent (Invitrogen, Carlsbad, CA, USA) according to the manufacturer’s instructions. For cDNA synthesis, 1 µg of total RNA was mixed with oligo dT primers using Maxime RT premix (iNtRON Biotechnology, Sungnam, Korea) and water to a total volume of 20 µL and incubated. The PCR amplification conditions were as follows: IL-1β, 95 °C, 30 s, 54 °C, 30 s, 72 °C, 60 s for a total of 35 cycles; IL-6, 95 °C, 30 s, 57 °C, 30 s, 72 °C, 60 s for a total of 35 cycles; ICAM-1, 95 °C, 30 s, 57 °C, 30 s, 72 °C, 60 s for a total of 35 cycles; TARC, 95 °C, 30 s, 54 °C, 30 s, 72 °C, 60 s for a total of 35 cycles; MDC, 95 °C, 30 s, 65 °C, 30 s, 72 °C, 60 s for a total of 35 cycles; β-actin, 95 °C, 30 s, 60 °C, 30 s, 72 °C, 60 s for a total of 35 cycles. The primer sequences are shown in Table 1.

### 2.6. Enzyme-Linked Immunosorbent Assay (ELISA)

HaCaT cells (1 × 10^6^ cells/well) were treated with TNF-α/IFN-γ or with medium alone in the presence or absence of NS or FS for 24 h. The culture medium was harvested and transferred into an ELISA plate. The IL-1β (DLB50; range 3.9–250 pg/mL), IL-6 (6050; range 3.1–800 pg/mL), TARC (DDN00; range 31.2–2000 pg/mL), and MDC (DMD00; range 125.0–4000 pg/mL) produced were measured by an ELISA kit (R&D system), according to the manufacturer’s instructions.

### 2.7. Western Blot Analysis

HaCaT cells (1 × 10^6^ cells/well) were treated with TNF-α/IFN-γ or with medium alone in the presence or absence of NS or FS for 30 min for the detection of IκB-α, phospho-NF-κB p65, and STAT1. The cells were washed twice with PBS and lysed in a lysis buffer (150 mM NaCl, 50 mM Tris-HCl, 1% Trizol, 0.1% SDS, 0.25% sodium deoxycholate, and 2-mercaptoethanol) containing protease and phosphatase inhibitors. Equal amounts of protein extracts (40 µg) were subjected to 10% SDS-PAGE, and the resultant gel was transferred to a PVDF membrane. The membrane was incubated with TBS-T buffer (pH 7.5, 150 mM NaCl, 50 mM Tris-HCl, and 0.1% Tween 20) containing 5% skim milk for 1 h, followed by incubation at 4 °C overnight with the primary antibodies. Subsequently, the membrane was incubated with the secondary antibodies for 1 h at room temperature. The membrane was washed three times with TBS-T, and color was detected by the PowerOpti-ECL western blotting detection reagent (Bionete, Hwaseong, Korea). The images were captured using the ChemiDoc imaging software (Bio-Rad, Hercules, CA, USA).

### 2.8. Statistical Analysis

All data are presented as means ± SD (standard deviation) of samples in triplicated samples. The values were analyzed through one-way ANOVA followed by Duncan’s multiple range tests using SAS 9.4 (SAS Institute Inc., Cary, NC, USA). The differences were considered statistically significant if *p* < 0.05.

## 3. Results and Disscusion

### 3.1. Changes in Protein Distribution

**Figure 1:**
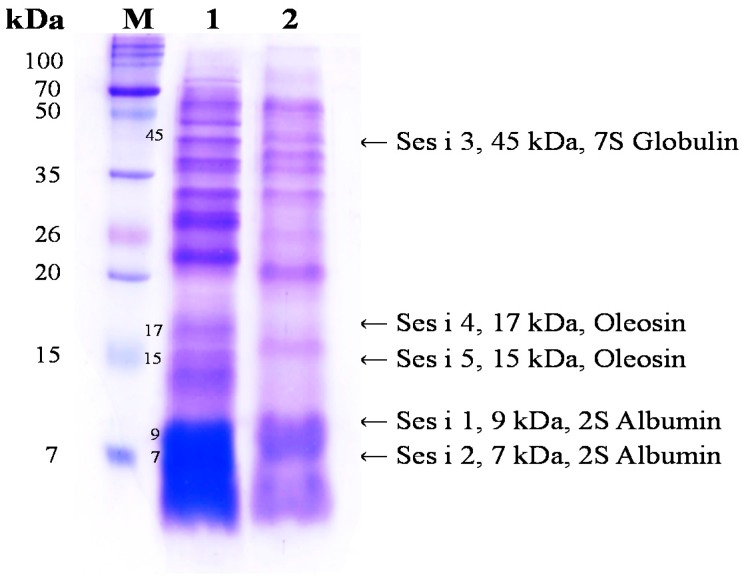
SDS-PAGE analysis of non-fermented sesame (NS) and fermented sesame (FS) proteins. Abbreviations: M, molecular weight standard maker; Lane 1, non-fermented sesame; Lane 2, fermented sesame.

Figure 1 shows the results of the electrophoresis of NS and FS, which are indicative of the protein distribution. The electrophoretic profile of non-fermented sesame (Lane 1) presented high-intensity bands of 2S albumin subunits (7, 9 kDA, Ses i 1, 2), 7S globulin (45 kDa, Ses i 3), and oleosin subunits (15, 17 kDa, Ses i 4, 5). Currently, the five major allergens of sesame include 2S albumin and oleosin subunits. The electrophoretic pattern of fermented sesame (lane 2) showed the degradation of all major allergen protein bands. Similarly, Meinlschmidt et al. [17] reported the degradation of major soybean allergens following fermentation with *Bacillus subtilis* and *Lactobacillus helveticus*. In addition, the degradation of soybean allergen during fermentation with microbial protease in fermented soybean food has been reported [18]. These results suggest that after fermentation, sesame allergens are degraded into small peptides. Food allergic reactions cause a variety of symptoms associated with the skin and respiratory system. The immunoglobulin E-mediated response has an important role in the pathogenesis of AD. Recent studies have reported the control of allergens through food processing by methods such as genetic modification and heat treatment. However, the sesame allergen Ses i 1 is known to be structurally very stable and is not degraded by heat treatment. Additionally, Moreno et al. [19] reported that Ses i 1 could sufficiently retain its three-dimensional structure while surviving the degradative environment of the gastrointestinal tract in order to sensitize an individual, enhancing the general immunogenicity and provoking an allergic reaction in the sensitized individual. Therefore, sesame fermentation not only enhances antioxidant activities and bioactive compounds, but also effectively reduces allergenicity.

### 3.2. Cell Viability

**Figure 2:**
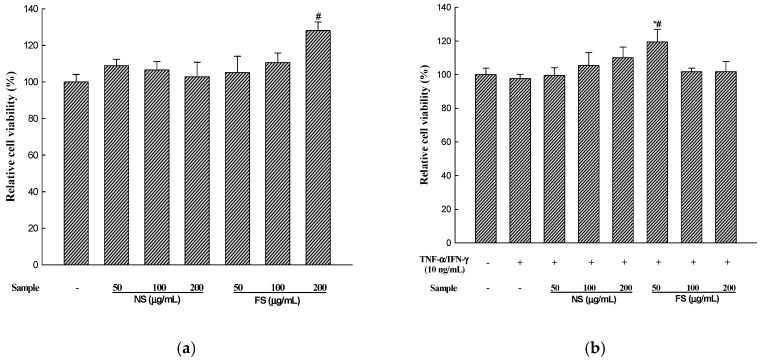
Effects of NS and FS extracts on cell viability in HaCaT cells. The cells were pre-incubated for 24 h, and cell viability was determined after treatment with NS and FS for 24 h (**a**); Cell viability after treatment with TNF-α/IFN-γ (10 ng/mL) in the presence of NS and FS (50, 100, 200 µg/mL) was also determined (**b**). Each bar represents the mean ± SD of triplicate determinations, *n* = 3; # *p* < 0.05 vs. vehicle controls; * *p* < 0.05, ** *p* < 0.01, *** *p* < 0.001 vs. TNF-α/IFN-γ treatment alone.

The compound XTT is cleaved by the mitochondrial dehydrogenase enzyme to produce a dark colored formazan in living cells with active metabolism [20]. To determine the effect of various concentrations of non-fermented sesame (NS) and fermented sesame (FS) on HaCaT cell viability, the XTT assay was used. Figure 2 shows the viability of HaCaT cells after using different concentrations of NS and FS. In comparison with the control group, the viability of the cells in the presence of NS ranged from 102.83 to 108.94% and that in the presence of FS ranged from 105.22 to 128.13%, respectively. NS and FS showed no cytotoxic effects on HaCaT cells. In addition, cytotoxicity was not observed in the HaCaT cells treated with TNF-α/IFN-γ and NS or FS. Therefore, we used 200 µg/mL of NS and FS in subsequent experiments.

### 3.3. mRNA Expressions of IL-1β, IL-6, ICAM-1, TARC, and MDC

**Figure 3:**
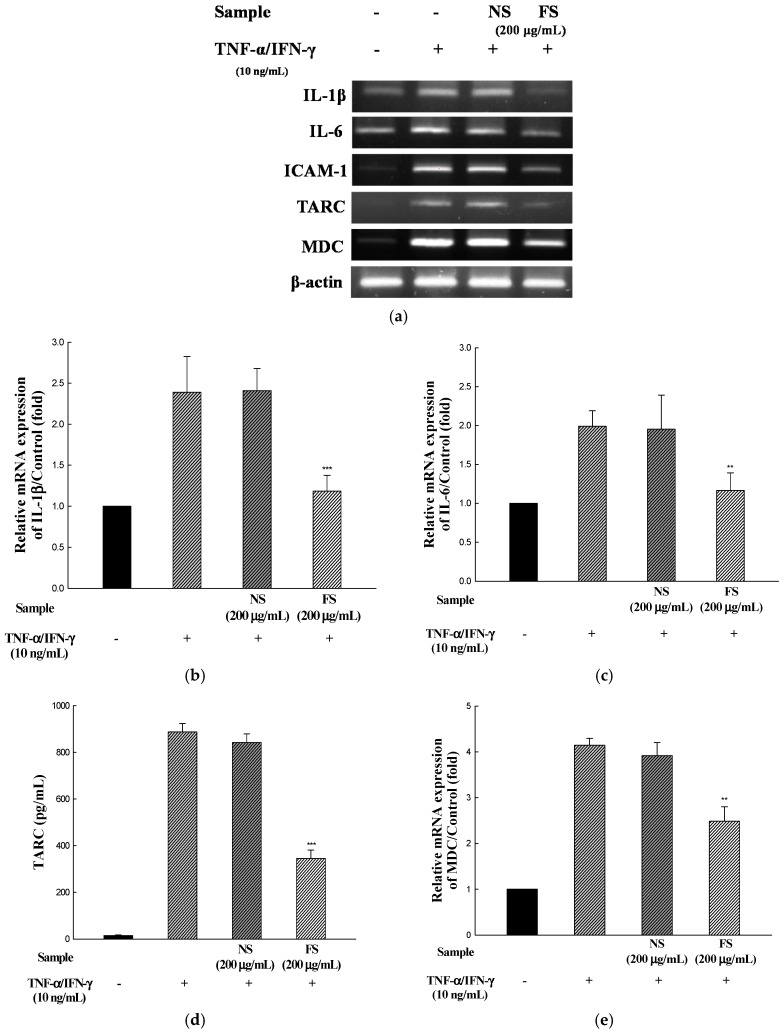

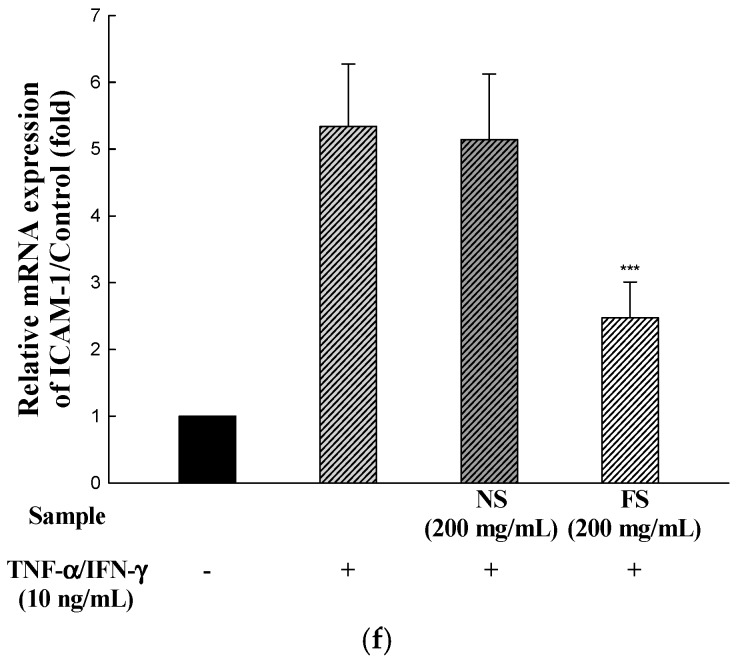
Effects of NS and FS extracts on TNF-α/IFN-γ-induced mRNA expression in HaCaT cells. RT-PCR was performed to determine the mRNA expression levels of IL-1β, IL-6, ICAM-1, TARC, and MDC (**a**); The intensities of the PCR bands for IL-1β (**b**); IL-6 (**c**); ICAM-1 (**d**); TARC (**e**); MDC (**f**). Each bar represents the mean ± SD of triplicate determinations, *n* = 3; * *p* < 0.05, ** *p* < 0.01, *** *p* < 0.001 vs. TNF-α /IFN-γ treatment alone. +, TNF-α/IFN-γ treatment; −, TNF-α/IFN-γ untreated.

To determine the inhibitory effects of NS and FS on pro-inflammatory cytokine and chemokine mRNA levels, human keratinocyte HaCaT cells were incubated for 24 h with TNF-α/IFN-γ in the presence of NS and FS. As shown in Figure 3a–f, the levels of IL-1β, IL-6, ICAM-1, TARC, and MDC mRNAs were increased by TNF-α/IFN-γ stimulation. The increase in the levels was inhibited following FS treatment. By contrast, NS did not have inhibitory effects on IL-1β, IL-6, ICAM-1, TARC, and MDC. The stimulation of HaCaT cells with TNF-α/IFN-γ induces the expression of pro-inflammatory cytokines and chemokines.

Chemokines are important in the induction of the inflammatory reaction and immune response. In AD patients, the infiltration of Th2-type lymphocytes into skin lesions is associated with high-level expression of chemokines such as MDC and TARC [21].

TARC is a chemokine involved in white blood cell infiltration into the skin. CCL17 is considered a pivotal mediator in the inflammatory responses during the development of Th2-dominant inflammatory skin diseases such as AD [9]. MDC is a chemokine that potently serves as a chemoattractant for monocytes, monocyte-derived dendritic cells, and natural killer cells [22]. The ligands for CCR4 are TARC and MDC, whose levels in the blood of AD patients are closely related to the levels observed during the onset of AD. In addition, the upregulation of ICAM-1 expression in keratinocytes has been observed in several inflammatory dermatoses, such as psoriasis, atopic dermatitis, and lupus erythematosus [23].

Several researchers have reported that the biological activity of natural products might be increased by fermentation. Lee et al. [24] reported that fermented soybean showed higher anti-inflammatory effects on the skin than non-fermented soybean.

### 3.4. IL-1β, IL-6, TARC, and MDC Production

**Figure 4:**
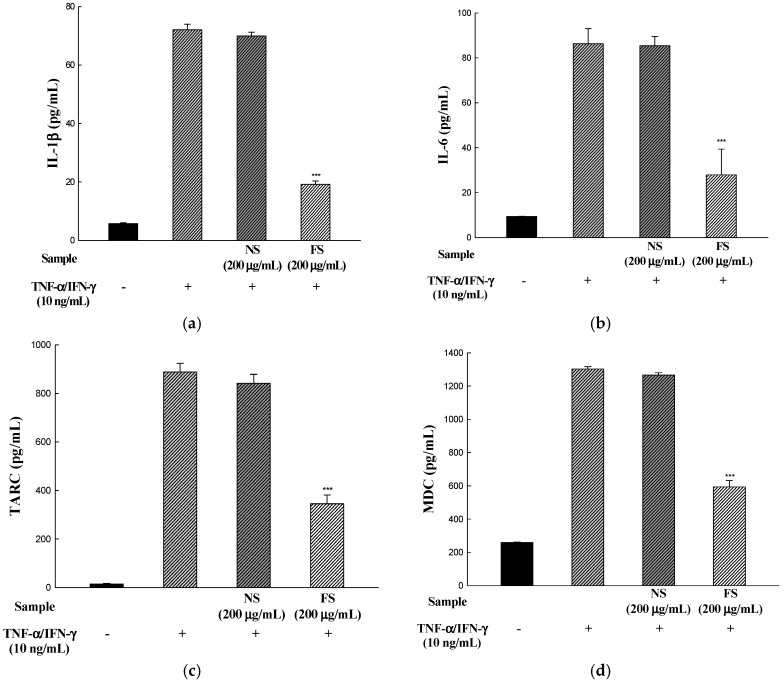
Effects of NS and FS extracts on TNF-α/IFN-γ-induced IL-1β, IL-6, TARC, and MDC production in HaCaT cells. The production of IL-1β (**a**); IL-6 (**b**); TARC (**c**); MDC (**d**) protein was measured by ELISA. Each bar represents the mean ± SD of triplicate determinations, *n* = 3; * *p* < 0.05, ** *p* < 0.01, *** *p* < 0.001 vs. TNF-α/IFN-γ treatment alone. +, TNF-α/IFN-γ treatment; −, TNF-α/IFN-γ untreated.

To determine whether NS and FS inhibit TNF-α/IFN-γ-stimulated IL-1β, IL-6, TARC, and MDC production, HaCaT cells were incubated for 24 h with TNF-α/IFN-γ in the presence of NS and FS, and culture medium supernatants were measured for these pro-inflammatory cytokines and chemokines with an ELISA kit. FS decreased TNF-α/IFN-γ-induced protein expression of IL-1β, IL-6, TARC, and MDC. In contrast, NS did not inhibit IL-1β, IL-6, TARC, and MDC protein expression, similar to mRNA expression (Figure 4a–d).

IL-6 is known to be acutely expressed at the early stage of inflammation and causes chronic inflammation when its secretion persists. Studies in peritonitis models suggest that IL-6 signaling is of crucial importance in the transition from the acute to the chronic phases of inflammatory processes [25]. In a normal patch test with haptens lesions, the pro-inflammatory cytokine IL-1β is the first detectable cytokine, which induces the migration of Langerhans cells from the epidermal compartment [26]. The pro-inflammatory cytokines IL-6 and IL-1β stimulate keratinocyte proliferation and leukocyte migration. Therefore, FS may regulate the infiltration of Th2 cells into the lesions of the skin by inhibiting mRNA expression and protein production of pro-inflammatory cytokines and chemokines related to AD.

### 3.5. Activation of STAT1 and NF-κB

**Figure 5:**
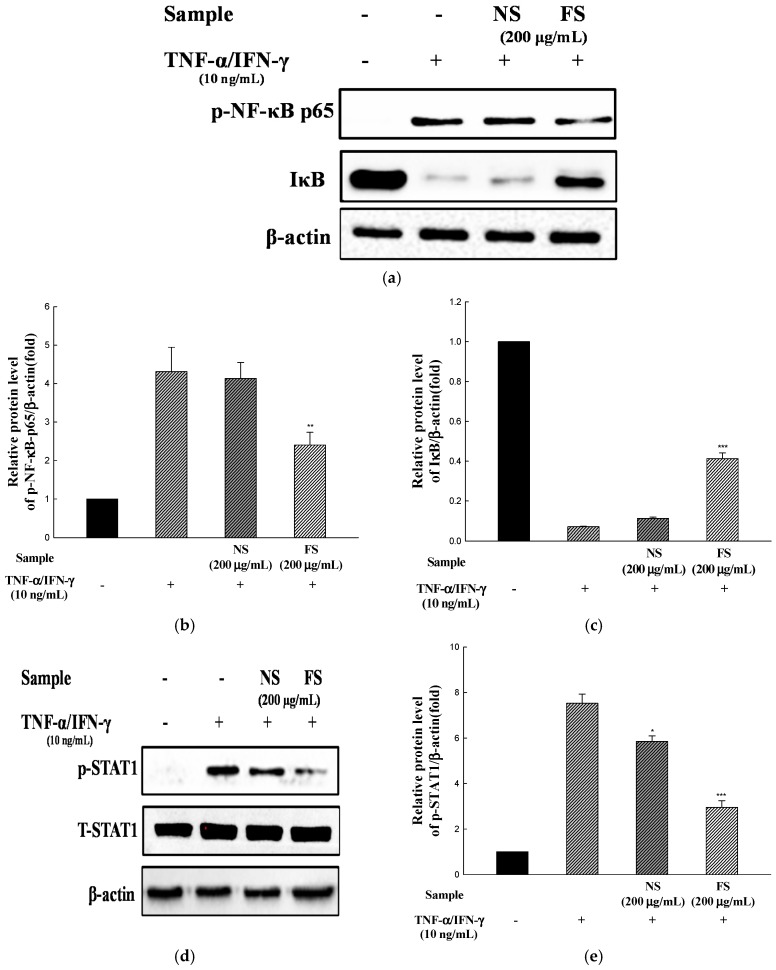
Effects of NS and FS extracts on TNF-α/IFN-γ-induced NF-κB and STAT1 activation in HaCaT cells. Total protein expression and phospho-NF-κB and IκB were examined by western blot (**a**); The relative protein level was calculated for the p-NF-κB/β-actin (**b**); The relative protein level was calculated for the IκB/β-actin (**c**); Total protein expression and phosphorylation of STAT1 were examined by western blot (**d**) and the relative protein level was calculated for p-STAT1/β-actin (**e**). Each bar represents the mean ± SD of triplicate determinations, *n* = 3; * *p* < 0.05, ** *p* < 0.01, *** *p* < 0.001 vs. TNF-α/IFN-γ treatment alone. +, TNF-α/IFN-γ treatment; −, TNF-α/IFN-γ untreated.

We investigated the effects of NS and FS on the activation of NF-κB and STAT1. The cells were incubated for 30 min with TNF-α /IFN-γ in the presence of NS and FS and then analyzed by western blotting. FS inhibited the phosphorylation of NF-κB p65 and the degradation of IκB-α (Figure 5a), but NS did not show any inhibitory effects. Likewise, FS inhibited the TNF-α/IFN-γ-induced activation of STAT1 (Figure 5d).

TARC and MDC promoters contain STAT1 and NF-κB-binding sequences and these transcription factors may mediate the transcription of these genes [27]. NF-κB is a transcription factor that regulates the transcription of genes that are important in the occurrence of inflammatory diseases. Phosphorylation of IκB-α by IκB-α kinase (IKK) triggers IκB-α ubiquitinylation and degradation, which liberates NF-κB, allowing it to translocate to the nucleus [28]. STAT1 activation is regulated by the activation of Janus Kinase (JAK). IFN-γ activates JAK1/2, which then phosphorylate STAT1 protein [29]. The phosphorylated STAT1 protein is translocated to the nucleus and activates the expression of chemokines such as TARC and MDC. Previous studies showed that NF-κB and JAK/STAT signaling pathways were involved in the regulation of pro-inflammatory cytokine, chemokine, and adhesion molecule production in HaCaT cells [30].

These results indicate that the inhibition of TNF-α/IFN-γ-induced pro-inflammatory cytokine and chemokine production by FS occurred through the suppression of NF-kB and STAT1 activation. Ju et al. [10] reported that the treatment with 1,2,3,4,6-Penta-*O*-galloyl-β-d-glucose alone did not affect mRNA expression, NF-κB, and STAT1 activation in HaCaT cells. In this study, the effects of NS and FS alone on cytokine, chemokine expression, and signaling pathways were not discussed. However, our previous studies showed that antioxidant activity and the amount of bioactive compounds of sesame were increased by bioconversion [13]. Further study is needed to determine the effect of FS treatment alone on TNF-α/IFN-γ action.

## 4. Conclusions

In summary, this study evaluated the changes in the anti-allergic activities of NS and FS by bioconversion. The electrophoresis patterns of FS demonstrated the degradation of all major allergen protein bands. FS decreased the protein and mRNA expression of IL-1β, IL-6, TARC, and MDC. In contrast, NS did not inhibit the protein and mRNA expression of IL-1β, IL-6, TARC, and MDC. We demonstrated that the FS exerts anti-allergic activities by suppressing pro-inflammatory cytokines (IL-1β, IL-6) and chemokines (TARC, MDC) through blockade of the phosphorylation of the NF-κB and STAT1 signaling pathways in HaCaT cells. Thus, fermented sesame products could be developed as a functional food derived from a natural product.

## Figures and Tables

**Table 1:** Reverse Transcription-Polymerase Chain Reaction (RT-PCR) primer sequences.

Genes	Forward	Reverse	Length (bp)
IL-1β	AAAAGCTTGGTGATGTCTGG	TTTCAACACGCAGGACAGG	176
IL-6	AGAGTAACTGAGGAACAAGCC	TACATTTGCCGAAGAGCCCT	238
MDC	AGGACAGAGCATGGCTCGCCTACAGA	TAATGGCAGGGAGGTAGCGCTCCTGA	361
TARC	CTTCTCTGCAGCACATCC	AAGACCTCTCAAGGCTTTG	236
ICAM-1	CACCCTAGAGCCAAGGTGAC	CATTGGACTCTGCTGGGAAT	251
β-Actin	GCGGGAAATCGTGCGTGACATT	GATGGAGTTGAAGGTAGTTTCGTG	231
